# Genetic variation analysis of reemerging *porcine epidemic diarrhea virus* prevailing in central China from 2010 to 2011

**DOI:** 10.1007/s11262-012-0867-x

**Published:** 2012-12-27

**Authors:** Xia Yang, Jin-yao Huo, Lu Chen, Feng-mei Zheng, Hong-tao Chang, Jun Zhao, Xin-wei Wang, Chuan-qing Wang

**Affiliations:** College of Animal Science and Veterinary Medicine, Henan Agricultural University, Zhengzhou, 450002 China

**Keywords:** *Porcine epidemic diarrhea virus*, Molecular epidemiology, Phylogenetic analysis, *M* gene, *ORF3* gene

## Abstract

Porcine epidemic diarrhea has re-emerged with devastating impact in central China since October 2010. To investigate and analyze the reason of this outbreak, the *M* and *ORF3* genes of 15 *porcine epidemic diarrhea viruses* (PEDV), which were collected from different areas of central China during October 2010 and December 2011, were amplified by reverse transcriptase polymerase chain reaction, cloned, sequenced, and analyzed. Sequence analyses showed that the nucleotides and amino acids were changed at some sites in the *M* and *ORF3* genes of the 15 PEDV strains compared with those genes of CV777 reference strain. Based on the phylogenetic analyses, PEDVs in central China and reference strains could be separated into three groups: G1, G2, and G3. The 15 PEDV strains belonged to G3 group and showed a close relationship with Korean strains (2007), Thai strains (2007–2008), and partial other Chinese strains (2010–2011), but differed genetically from European strains (Br1/87) and the vaccine strain (CV777 vs) being used in China. Furthermore, all 15 PEDV strains from central China and some other isolates in China from 2003 to 2007 (LJB-03, QH, and LZC) belonged to different group. Therefore, PEDV exhibits rapid variation and genetic evolution, and the currently prevailing PEDV strains in central China are a new genotype.

## Introduction

*Porcine epidemic diarrhea virus* (PEDV), a member of family Coronaviridae, is an enveloped, single-stranded, positive-sense RNA virus [1]. The complete genome sequence of PEDV is approximately 28 kb, including a 5′ untranslated region (UTR), a 3′ UTR and at least seven open reading frames (ORFs): ORF1a, ORF1b, and ORF2-6 [2]. The 5′ two-thirds of the genome are occupied by ORF1a and ORF1b, which encode nonstructural proteins. The 3′ one-third of the genome contains genes which encode the spike (S, 150–220 kDa), envelope (E, 7 kDa), membrane (M, 20–30 kDa), and nucleoprotein (N, 58 kDa) separately. There is also a nonstructural *ORF3* gene between S- and E-protein encoding regions.

PEDV can cause porcine epidemic diarrhea (PED), an acute and highly contagious enteric disease characterized by severe enteritis, vomiting, watery diarrhea, and dehydration with high mortality in suckling piglets. PED was first reported in Belgium [3] and the United Kingdom [4] in 1978. It has subsequently been reported in many other swine-producing countries throughout the world. The presence of PED in China was confirmed by the immunofluorescence assay and serum neutralization test in 1984 [5]. PED has spread and become widely prevalent in some countries and regions of Asia in recent years [6–10]. It also outbroke in China during 2010–2011 [11–13]. Pigs of all ages exhibited especially severe diarrhea and dehydration with up to 100 % mortality among suckling piglets. The pigs of Henan, Shanxi, Anhui, and Hebei provinces located in central China, of which breeding stock accounts for 25 % of the total pig population in China, were seriously affected and sustained great losses. Porcine intestinal tissues and fecal specimens were collected between October 2010 and December 2011 from 82 swine farms in the four provinces in central China. Among those farms, 51 samples were confirmed to be positive for PEDV by reverse transcriptase polymerase chain reaction (RT-PCR). In the present study, 15 representative samples from different area at different time were selected from 51 positive samples to investigate the molecular epidemiology and gene variation of PEDV in central China using sequence analyses of the *M* and *ORF3* genes so as to determine the reason for the recurrence of this disease and provide recommendations for its prevention.

## Materials and methods

### Positive samples

15 intestinal tissues and fecal specimens were collected from four provinces located in central China from October 2010 to December 2011, which were determined to be positive for PEDV by RT-PCR and excluded the contaminant of extraneous *transmissible gastroenteritis virus*, *rotavirus* *A*, *kobuvirus*, *porcine circovirus*-*2*, and *suid herpesvirus 1* by PCR or RT-PCR assay. The detailed informations of PEDV strains are shown in Table 1.

**Table 1:** PEDV strains for sequence alignment and phylogenetic analysis

Isolate	Accession number	Origin	Isolate	Accession number	Origin
**CH/ZMDZY/11** ^**M/O**^	JN400898/JN547391	China, 2011	QH^M^	AY974335	China, 2005
**CH/XXHJ/11** ^**M/O**^	JN400899/JN547392	China, 2011	LJB-03^M^	AY608890	China, 2003
**CH/XCYL/11** ^**M/O**^	JN400900/JN547393	China, 2011	CPF531^M^	FJ687471	Korea, 2007
**CH/ZKHFQ/11** ^**M/O**^	JN400901/JN547394	China, 2011	M2227^M^	FJ687456	Korea, 2004
**CH/PDSBF/11** ^**M/O**^	JN400903/JN547396	China, 2011	M1595^M^	FJ687452	Korea, 2003
**CH/KFQX/11** ^**M/O**^	JN400904/JN547397	China, 2011	KPEDV-9^M^	AF015888	Korea, 1997
**CH/XCYL2/11** ^**M/O**^	JN584167/JN584172	China, 2011	VN103M2^M^	HQ883480	Vietnam, 2010
**CH/PDSXX/11** ^**M/O**^	JN400905/JN547398	China, 2011	08RB03^M^	FJ196184	Thailand, 2008
**CH/ZKFG/11** ^**M/O**^	JN400906/JN547399	China, 2011	M_NIAH116099_07^M^	EU542417	Thailand, 2007
**CH/ZKXH/11** ^**M/O**^	JN400907/JN547400	China, 2011	M_NIAH1795_04^M^	EU542415	Thailand, 2004
**CH/XXYY/11** ^**M/O**^	JN400908/JN547401	China, 2011	M_NIAH380_98^M^	EU581712	Thailand, 1998
**CH/HBXX2/11** ^**M/O**^	JN584168/JN584170	China, 2011	M_NIAH2013_95^M^	EU581711	Thailand, 1995
**CH/NYWL/11** ^**M/O**^	JN584169/JN584171	China, 2011	JMe2^M^	D89752.1	Japan, 1996
**CH/HBXX3/11** ^**M/O**^	JQ664730/JQ664731	China, 2011	CH/XJUrumqi/2011^O^	JQ027027	China, 2011
**CH/HBQX/10** ^**M/O**^	JN400902/JN547395	China, 2010	CH/FJND-1/2011^O^	JQ027021	China, 2011
GDYD^M^	JN089731	China, 2011	CHGD-01^O^	JN980697	China, 2011
GDYA^M^	JN089729	China, 2011	CH/HLJHRB/2011^O^	JQ027026	China, 2011
QYA1^M^	JN089742	China, 2011	CV777 Vaccine Strain^O^	GU372744	China, 2009
DBA1^M^	JN083782	China, 2011	CH/JL/09^O^	GU372741	China, 2009
HBQY^M^	JN400911	China, 2011	CH/JL/08^O^	GU372734	China, 2008
HB/FN^M^	JF508465	China, 2010	CH/GSJI/07^O^	GU372737	China, 2007
GDST1^M^	JN083788	China, 2010	CH/GSJIII/07^O^	GU372743	China, 2007
BJ2010^M^	JF690778	China, 2010	CH/SHH/06^O^	GU372740	China, 2006
DX^M^	EU031893	China, 2007	CHIMT06^O^	GU372739	China, 2006
LZC^M/O^	EF185992/EF185992	China, 2006	CH/S^O^	GU372733	China, 1986
CPF299^M/O^	FJ687467/HQ537450	Korea, 2007	VF131^O^	HQ537444	Korea, 2007
V2501^M/O^	FJ687458/HQ537441	Korea, 2005	DR13^O^	EU054929	Korea, 2007
Chinju99^M/O^	DQ845249/EU792474	Korea, 1999	M2366^O^	HQ537440	Korea, 2004
Br1/87^M/O^	Z24733/Z24733	Britain, 1987	BI976^O^	HQ537433	Korea, 2003
CV777^M/O^	AF353511/AF353511	Belgium, 1993	DBI865^O^	HQ537432	Korea, 2002

^M^ *M* gene used for sequence analysis, ^O^ *ORF3* gene used for sequence analysis, Bold text in the table means 15 strains sequenced in this study

### Primers

Two sets of primers were designed and synthesized (Sangon biotech, China) to amplify the *M* and *ORF3* genes, respectively (Table 2), based on the genome of PEDV CV777 (GenBank No. AF353511).

**Table 2:** Primers used for amplifying *M* and *ORF3* genes of PEDV

Name of primer	Forward/reverse	Primer sequence (5′–3′)	Target gene	Fragment size (bp)	Genomic co-ordinates
M-F	Forward	TACATGCGAATTGACCCCCT	*M*	873	CV777strain (AF353511)
M-R	Reverse	ATCCTTGTTAGTGGGTACAGCG			
O-F	Forward	CGTGGTGGGTTTGGTTGAT	*ORF3*	964	
O-R	Reverse	CGGTGACAAGTGAAGCACAGAT			

### Sample preparation

Intestinal tract and fecal specimens of sick pigs were diluted five times in phosphate buffered saline. The samples were ground and then centrifuged at 8,000×*g* for 10 min after freezing and thawing three times. The supernatants were stored at −20 °C.

### RNA extraction

PEDV RNA was extracted and dissolved in 30 μl 1 % diethyl pyrocarbonate water, as described in the Trizol reagent introduction (Invitrogen), then stored at −70 °C.

### Amplification of *M* and *ORF3* gene

The 20-μl reverse transcription system consisted of 13 μl purified RNA, 4 μl 5× buffer (50 mM Tris–HCl, 75 mM KCl, 3 mM MgCl_2_, 10 mM DTT), 10 mmol dNTP, 20 pmol random primer, 20 U RNase inhibitor, and 100 U reverse transcriptase M-MLV. The assay was reacted at 42 °C for 1.5 h, then at 95 °C for 5 min.

The PCR mixtures consisted of 5 μl 10× Taq DNA polymerase buffer (10 mM Tris-HCl, 50 mM KCl), 2.5 mmol MgCl_2_, 5 μl cDNA, 10 mmol dNTP, 2.5 U Taq DNA polymerase, and 20 pmol each sense and antisense specific primer of *M* or *ORF3* gene. Sterile distilled H_2_O was added to reach a total volume of 50 μl. Amplification was performed as follows: one cycle at 95 °C for 5 min followed by 32 cycles at 95 °C for 1 min, 55 °C for 1 min, 72 °C for 1.5 min, and a final extension at 72 °C for 10 min. The products were stored at 4 °C.

### Cloning and sequencing of PCR products

PCR products were identified by electrophoresis on 1 % agarose gel and cloned into pMD18-T. The recombinant vector was identified by PCR and enzyme digestion. The positive clones were sent to Beijing Genomics Institute (BGI) for sequencing. All sequencing reactions were performed in duplicate. The sizes of PCR products should be 873 and 964 bp, respectively. The former contained the *M* gene and its flanking sequences, while the latter contained *ORF3* gene and its flanking sequences.

### Sequence analysis of *M* and *ORF3* genes

Nucleotide and the deduced amino acid sequences of *M* and *ORF3* genes of 15 strains were aligned and analyzed by the MegAlign software (DNAstar). The reference strains used for sequence alignment and analysis with the 15 PEDV strains are presented in Table 1.

### Phylogenetic analysis of PEDV

Phylogenetic trees were constructed by Molecular Evolutionary Genetics Analysis (MEGA) software (version 4.0) with the neighbor-joining method based on amino acid sequence of M and ORF3 proteins, respectively. To assess the relative support for each clade, bootstrap values were calculated from 1,000 replicate analyses. The reference strains used for phylogenetic analysis with the 15 PEDV strains are presented in Table 1.

## Results

### Sequencing of the *M* and *ORF3* genes

All the *M* and *ORF3* genes of the PEDV strains from 15 farms were amplified and sequenced. The sequences were submitted to GenBank. PEDV strains sequenced in this study and their accession numbers are shown in bold text in Table 1.

### Nucleotide and deduced amino acid sequence analyses of the *M* gene

The length of the *M* gene of all 15 PEDV strains from central China was 681 nucleotides encoding a protein of 226 amino acids with a predicted molecular weight of 25.3–25.4 kDa. Based on the sequence analysis and comparison with CV777 reference strain, the following nucleotide changes were observed: G→C at 49; T→C at 137, 297 and 630; G→A at 192; G→T at 210 and 652; C→T at 225, 234 and 246; T→A at 360; and A→C at 615. Furthermore, two other nucleotide changes in CH/XXYY/11, CH/XCYL2/11, CH/PDSBF/11, CH/XXHJ/11, CH/KFQX/11, CH/ZKFG/11, CH/XCYL/11, and CH/NYWL/11 strains were observed: A→T at 50 and A→G at 73. These specific nucleotide changes led to amino acid changes at position 46 (V→A) and 218 (A→S). For the CH/XXYY/11, CH/XCYL2/11, CH/PDSBF/11, CH/XXHJ/11, CH/KFQX/11, CH/ZKFG/11, CH/XCYL/11, and CH/NYWL/11 strains, the additional amino acid changes at position 13 (E→L, the other seven isolates underwent E→Q at this site) and 25 (T→A) were observed.

Results of sequence identity based on the complete *M* gene of all PEDV strains are shown precisely in Table 3. The results revealed that 15 PEDV strains from central China shared the highest identity with Group 3-4, which consisted of six Chinese strains (HB/FN, DX, BJ2010, GDYA, HB/QY, GDST1) from 2007 to 2011, two Thai strains (08RB03, M_NIAH116099_07) from 2007 to 2008 and Korean CPF299 strain in 2007 at nucleotide and amino acid level.

**Table 3:** Nucleotide and deduced amino acid sequence identity based on *M* genes of PEDV strain from central China with reference strains (%)

Group	15 strains^a^	Group 1^b^	Group 2^c^	Group 3			
				Group 3-1^d^	Group 3-2^e^	Group 3-3^f^	Group 3-4^g^
15 strains		95.9–98.8	96.5–98.4	96.9–98.2	98.4–99.1	97.2–99.6	97.8–100
Group 1	94.7–98.7		96.8–98.5	96.0–98.2	96.6–99.3	96.5–98.6	96.1–98.8
Group 2	95.6–88.7	95.6–99.1		96.9–98.5	97.2–98.2	97.1–99.3	96.7–98.8
Group 3							
Group 3-1	95.6–98.7	95.2–98.2	96.0–99.1		97.7–98.2	97.4–98.4	97.2–98.2
Group 3-2	97.4–99.1	96.9–99.6	96.9–98.7	96.9–98.7		97.9–99.0	98.5–99.1
Group 3-3	96.5–99.6	95.2–99.1	96.0–99.1	96.5–99.1	98.2–99.6		97.5–99.9
Group 3-4	97.8–100	95.6–98.7	96.4–98.7	96.9–98.7	98.7–99.1	97.8–99.6	

Data in upper right and lower left table fields indicate percentage of nucleotide and amino acid sequence similarity, respectively

^a^ 15 PEDV strains from central China in this study

^b^ Three Thai strains (M_NIAH1795_04, M_NIAH2013_95, M_NIAH380_98) from 1995 to 2004, Vietnam VN103M2 strain and Chinese DBA1 strain

^c^ European strains (CV777 and Br1/87) and Chinese LZC strain

^d^ Four Korean strains (CPF531, V2501, M2227, M1595) and two Chinese strains (QH, LJB/03) from 2003 to 2005

^e^ Chinese QYA1 strain

^f^ Two Korean strains (KPEDV-9, Chinju99) from 1997 to 1999, Chinese GDYD strain and Japanese JMe2 strain

^g^ Six Chinese strains (HB/FN, DX, BJ2010, GDYA, HB/QY, GDST1) from 2007 to 2011, two Thai strains (08RB03, M_NIAH116099_07) from 2007 to 2008 and Korean CPF299 strain

### Phylogenetic analysis based on the M protein

**Fig. 1:**
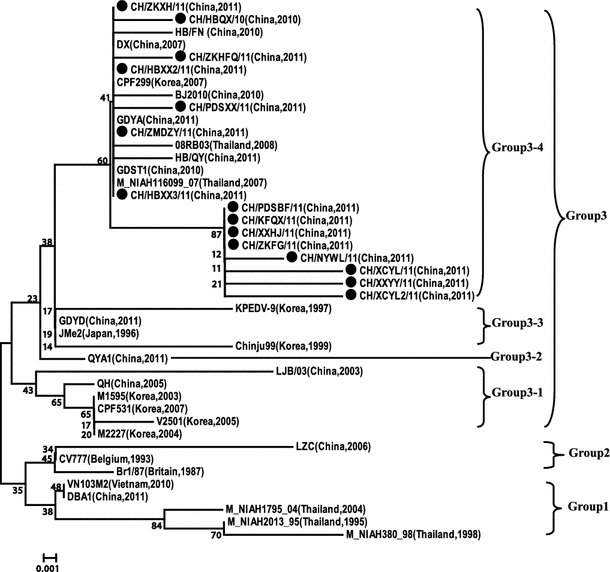
Phylogenetic analysis based on amino acid sequences of M protein of 15 PEDV strains from central China and reference strains. The 15 strains sequenced in this study are marked initially by *closed circle* symbols

Phylogenetic analyses based on the amino acid sequences of M protein showed that the PEDV strains fell into three groups (Fig. 1). Group 3 comprised four subgroups (3-1, 3-2, 3-3, and 3-4). All the strains in this study belonged to the subgroup 3-4, together with six other Chinese strains from 2007 to 2011, two Thai strains from 2007 to 2008, and a Korean strains (CPF299). The phylogenetic tree indicates that the 15 strains from central China have close relationship with the strains isolated from China, Thai and Korea after 2007.

### Nucleotide and deduced amino acid sequence analysis of the *ORF3* gene

The length of the *ORF3* gene of all 15 PEDV strains from central China was 675 nucleotides encoding a protein of 225 amino acids with a predicted molecular weight of 25.3–25.4 kDa. Based on the sequence analysis and comparison with CV777 reference strain, the following nucleotide changes were observed: G→A at 54, 160, 235, and 301; T→C at 62, 162, 360, 393, and 537; C→T at 63, 237, 243, 264, 274, 369, 439, and 450; T→G at 238 and 546; and A→G at 497. These specific nucleotide changes led to amino acid changes: V→A at 21, V→I at 54, V→I at 79, F→V at 80, L→F at 92, A→T at 101, N→S at 166, and H→Q at 182.

Results of sequence identity based on the complete *ORF3* gene of all PEDV strains are shown precisely in Table 4. The results showed that 15 PEDV strains from central China have 98.5–100 % (98.2–100 %) nucleotide (deduced amino acid) identity with each other and 97.3–99.6 % (97.3–100 %), 95.3–97.2 % (93.8–96.9 %), and 92.4–93.1 % (90.2–92.4 %) nucleotide (deduced amino acid) identity with members of group 3 (not included 15 PEDV strains from central China), 2 and 1, respectively. PEDVs of group 2 have 95.1–99.9 % (93.3–99.6 %) nucleotide (deduced amino acid) identity with each other and 90.6–92.0 % (89.1–91.3 %) nucleotide (deduced amino acid) identity with members of group 1. PEDVs of group 3 (not included 15 PEDV strains from central China) have nucleotide (deduced amino acid) identities of 97.3–100 % (97.3–100 %) nucleotide (deduced amino acid) identity with each other, and 95.3–97.8 % (94.2–97.3 %) and 92.0–93.5 % (90.2–92.4 %) nucleotide (deduced amino acid) identity with members of group 2 and 1. PEDVs of group 1 have 100 % (100 %) nucleotide (deduced amino acid) identity with each other.

**Table 4:** Nucleotide and deduced amino acid sequence identity based on *ORF3* genes of PEDV strain from central China with reference strains (%)

Group	15 strains^a^	Group 1^b^	Group 2^c^	Group 3^d^
15 strains		92.4–93.1	95.3–97.2	97.3–99.6
Group 1	90.2–92.4		90.6–92.0	92.0–93.5
Group 2	93.8–96.9	89.1–91.3		95.3–97.8
Group 3	97.3–100	90.2–92.4	94.2–97.3	

Data in upper right and lower left table fields indicate percentage of nucleotide and amino acid sequence similarity, respectively

^a^ 15 PEDV strains from central China

^b^ CV777 vaccine strains, Chinese CH/GSJIII/07 strain and Korean DBI865 strain

^c^ Two European strains (CV777, Br1/87) and two Chinese strains (LZC, CHGD-01) from 2006 to 2011

^d^ Nine Chinese strains (CH/GSJI/07, CH/FJND-1/2011, CH/SHH/06, CH/JL/09, CH/JL/08, CHIMT06, CH/XJUrumqi/2011, CH/HLJHRB/2011, CH/S) from 1986 to 2011 and seven Korean strains (Chinju99, VF131, DR13, BI976, V2501, M2366, CPF299) from 1999 to 2007

### Phylogenetic analysis based on the ORF3 protein

**Fig. 2:**
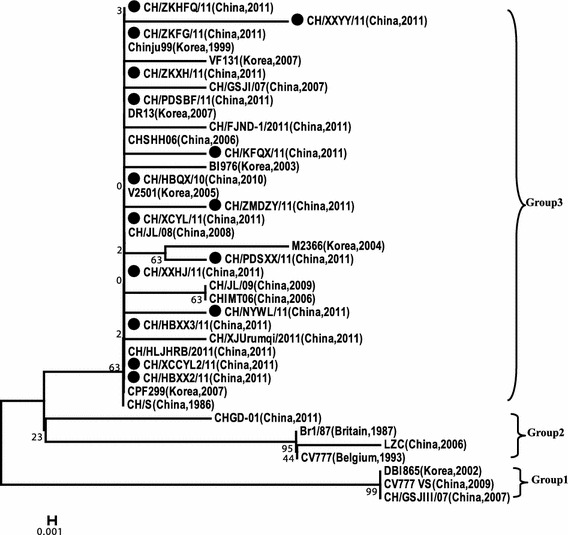
Phylogenetic analysis based on amino acid sequences of ORF3 protein of 15 PEDV strains in central China and reference strains. The 15 strains sequenced in this study are marked initially by *closed circle* symbols

Phylogenetic analysis showed that the PEDVs fell into three groups (Fig. 2). The 15 PEDV strains from central China belonged to group 3, together with nine isolates of other domestic strains from 1986 to 2011 and seven Korean strains from 1999 to 2007. Group 2 comprised a south China strain (CHGD-01) and two European strains (CV777 and Br1/87). Group 1 included a Korean strains (DBI865), CV777 vaccine strain (CV777 vs), and a Chinese strains (CH/GSJIII/07). They had distant relationship with CV777 vs.

## Discussion

The M protein encoded by the *ORF5* gene is a membrane glycoprotein. In addition to playing an important role in the viral assembly process [14], the M protein induces antibodies that can neutralize virus in the presence of complement. The nucleotide sequences of *M* gene possess low identity with other *coronavirus*, but are highly conserved among PEDV strains. Nonstructural proteins encoded by the *ORF3* gene of *coronavirus* closely relate to its pathogenicity. Highly adapted *transmissible gastroenteritis coronavirus* (TGEV) and PEDV have sequence variations in the *ORF3* gene and exhibit reduced pathogenicity [15, 16]. Wang et al. [17] has shown that the virulence to cells of PEDV was reduced by RNA silencing to inhibit the expression of *ORF3* gene. Therefore, the *ORF3* gene of PEDV has been suggested as an important virulence gene. The differences of *ORF3* genes between the wild-type and attenuated type can serve as a marker of the adaption to cells of PEDV and as a potential tool to study molecular epidemiology [16]. Therefore, the *M* and *ORF3* genes are often used to analyze the molecular epidemiology of PEDV [6, 10, 18].

A dual-combination, attenuated vaccine against TGEV and PEDV has been used in China since 1995 [19, 20]. This vaccine has significantly reduced the losses in the pig-breeding industry from these two diseases. However, according to the report of Chen et al. [6], PEDV became epidemic in immunized swine after 2006. Furthermore, as reported by Sun et al. [13], PED broke out in more than 10 provinces in South China and had caused more than one million piglets deaths since October 2010. Suspected PEDV-infected samples of feces and intestinal tissues, which were collected from 82 farms in Henan, Shanxi, Anhui, and Hebei provinces, were detected by duplex RT-PCR detecting TGEV and PEDV, which was developed in our laboratory. The result showed that the positive rate of PEDV was 62.2 % (51/82) and the positive rate of TGEV was 1.22 % (1/82). Furthermore, there was no co-infection of PEDV and TGEV (issued in another article). From these results, it can be deduced that the epidemic piglet diarrhea was mainly caused by single infection of PEDV, which was different from previous co-infections.

The *M* and *ORF3* genes of 15 PEDV strains were amplified by RT-PCR, cloned, sequenced, and analyzed to study the gene variation and molecular epidemiology of the PEDV that reappeared in central China and to determine the reason that the disease became epidemic again. Our study shows that there is a conserved ATAAAC sequence at the 11th nucleotide upstream of the initiation codon (ATG) of the *M* gene, and a conserved CTAGAC sequence at the 46th nucleotide upstream of the initiation codon (ATG) of *ORF3* gene which is consistent with previous reports [21]. Both sequences are hexameric motifs, similar to the six-dimerization motifs XUA (A/G) AC of other ORFs, which have been proven to be the transcription start sites of PEDV subgenomic mRNAs [22].

Based on the sequence analyses of the *M* and *ORF3* genes, amino acid changes occur on the *M* gene: V→A at 46 and A→S at 218. There are also amino acid changes on the *ORF3* gene: V→A at 21, V→I at 54, V→I at 79, F→V at 80, L→F at 92, A→T at 101, N→S at 166, and H→Q at 182. These changes of amino acids are consistent with those of the PEDV strains of China and its neighboring countries (Korea and Thailand) prevailing in recent years. Furthermore, nucleotides of the *M* genes of CH/XXYY/11, CH/XCYL2/11, CH/NYWL/11, CH/PDSBF/11, CH/XXHJ/11, CH/KFQX/11, CH/ZKFG/11, and CH/XCYL/11 change at 38 (A→T, amino acid change E→L at 13), 61 (A→G, amino acid change T→A at 21), which are different from previous strains. Amino acid changes of *M* gene, E→Q at 13, occur in seven isolates, are consistent with strains in China and neighboring countries. However, changes of the *M* gene in the rest of eight samples, resulting in amino acid change (E→L) at the same site, are different from other reference strains in China isolated in recent years. Therefore, PEDV strains which have prevailed in China in recent years are not exactly the same. There is a tendency toward variation instead.

Phylogenetic analysis based on the M protein shows that 15 PEDV strains have a close relationship with the other subgroup 3-4 members, including six isolates of other Chinese domestic strains (one from 2007, the others from 2010 to 2011), two Thai strains from 2007 to 2008 and a Korean strains (CPF299). Phylogenetic analyses based on the ORF3 protein show that the 15 PEDV strains have close relationships with nine isolates of other Chinese domestic strains and 7 Korean strains from 2007, but they differ genetically from the European strain and the CV777 vaccine strain (CV777 vs) used in China. In addition, CHGD-01 isolated from South China is closely related to the two European strains, which belong to the same group. The results show that PEDV in central China have high level of homology with other domestic PEDV strains in China from 2010 to 2011. It also has a close relationship with Korean strains from 2007 and Thai strains from 2007 to 2008. However, these 15 PEDV strains differ genetically from isolates in China from 2003 to 2006, two European strains (CV777 and Br1/87) and CV777 vs. It can be concluded that PEDV strains which were widely prevailing in central China from 2010 to 2011 exhibit rapid variation and evolution and belong to the same genotype, possibly traveling from neighboring countries.

Most of the samples in this study were selected from the farms where pigs were immunized with PED inactivated vaccine or attenuated vaccine, which were manufactured with CV777 vs. However, the morbidity and mortality of suckling piglets suffering from the PED outbreak were still high. The sample taken from a farm where severe diarrhea epidemic broke out in October 2011 was confirmed to be positive for PEDV. The infection at the farm subsided gradually by vaccine immunization and artificial infection of all the sows using the feces of sick pigs. But, PED broke out again at the same farm after only 2 months. PEDVs isolated from the farm at different time were named as CH/HBXX2/11 in October and CH/HBXX3/11 in December. The *M* and *ORF3* genes of the two PEDV strains were analyzed. Homology results show that the two PEDV strains have 100 % (100 %) nucleotide (deduced amino acid) sequence identities for the *M* gene and 99.9 % (100 %) nucleotide (deduced amino acid) sequence identities for the *ORF3* gene, which show that the two PEDV strains are the same strain. This result indicates that the current PEDV has strong immune escape capability or enhanced virulence, which can cause immunity failure. It is urgent to study the biologic characteristics, antigenicity, and mechanism of virulence variation of these strains further. Better vaccine strains must be developed for the prevention and control of the disease.
